# Abseq: Ultrahigh-throughput single cell protein profiling with droplet microfluidic barcoding

**DOI:** 10.1038/srep44447

**Published:** 2017-03-14

**Authors:** Payam Shahi, Samuel C. Kim, John R. Haliburton, Zev J. Gartner, Adam R. Abate

**Affiliations:** 1 Department of Bioengineering and Therapeutic Sciences and California Institute for Quantitative Biosciences (QB3), University of California, San Francisco, San Francisco, CA 94158, USA; 2 Department of Pharmaceutical Chemistry, University of California, San Francisco, San Francisco, CA 94158, USA

## Abstract

Proteins are the primary effectors of cellular function, including cellular metabolism, structural dynamics, and information processing. However, quantitative characterization of proteins at the single-cell level is challenging due to the tiny amount of protein available. Here, we present Abseq, a method to detect and quantitate proteins in single cells at ultrahigh throughput. Like flow and mass cytometry, Abseq uses specific antibodies to detect epitopes of interest; however, unlike these methods, antibodies are labeled with sequence tags that can be read out with microfluidic barcoding and DNA sequencing. We demonstrate this novel approach by characterizing surface proteins of different cell types at the single-cell level and distinguishing between the cells by their protein expression profiles. DNA-tagged antibodies provide multiple advantages for profiling proteins in single cells, including the ability to amplify low-abundance tags to make them detectable with sequencing, to use molecular indices for quantitative results, and essentially limitless multiplexing.

Proteins are the physical building blocks of cells, comprising the majority of cell mass and carrying out most cell functions, including cell structure dynamics, metabolism, and information processing. They are the molecular machines that convert thermodynamic potential into the energy of living systems. Measuring protein expression and modification is thus important for obtaining an accurate snapshot of cell state and function^1^,^2^,^3^,^4^. A common challenge when measuring proteins at the single-cell level is that most cell systems are heterogeneous, containing massive numbers of molecularly distinct cells^5^,^6^,^7^,^8^,^9^,^10^. A centimeter-sized tissue volume, for example, can contain billions of cells, each with its own unique spectrum of protein expression and modification; moreover, this underlying cellular heterogeneity can have important consequences on the system as a whole, such as in development, the regulation of the immune system, cancer progression and therapeutic response^11^,^12^,^13^,^14^. For heterogeneous systems like these, methods for high-throughput protein profiling in single cells are necessary.

Profiling proteins in single cells at high throughput requires methods that are sensitive and fast. Flow cytometry with fluorescently-labeled antibodies has been a bedrock in biology for decades because it can sensitively profile proteins in millions of single cells^15^,^16^. By labeling antibodies with dyes of different color, profiling can be multiplexed to tens of proteins^17^. By swapping dyes with mass tags and using a mass spectrometer for the readout, multiplexing can be increased to over a hundred antibodies^18^,^19^,^20^. Nevertheless, while these methods continue to improve in sensitivity and multiplexing, they remain far from enabling the characterization of the entire proteome in single cells, which for humans comprises >20,000 proteins and >100,000 epitopes^21^,^22^,^23^. A system that could sensitively profile all epitopes in a proteome would be extremely valuable, because it would obviate the need to select which proteins to target. However, existing methods with dye and mass tags are not scalable to the level of full proteome analysis, and in the case of mass-cytometry, destroy the transcriptome during analysis, making it challenging to obtain simultaneous measurements of proteome and transcriptome from the same single cell.

In this paper we present Abseq, a method to profile proteins in single cells that combines the speed of flow and mass cytometry with markedly increased sensitivity, accuracy, and multiplexing potential. In Abseq, the usual fluorophore or heavy metal-tagged antibodies are replaced with DNA sequence tags that can be read out at the single-cell level using droplet microfluidic barcoding^24^,^25^,^26^ and DNA sequencing. DNA tags afford a number of valuable advantages for labeling antibodies. They can be amplified from low levels to make them detectable with DNA sequencing, and can include unique molecular identifiers (UMIs) to correct for amplification bias and provide quantitative results^27^. Moreover, the tag identity is encoded by its full nucleobase sequence, providing a combinatorial tag space far exceeding what is possible with fluorescence or mass tags. For example, a modest tag length of ten bases provides over a million unique sequences, sufficient to label an antibody against every epitope in the human proteome. Indeed, with this approach, the limit to multiplexing is not the availability of unique tag sequences but, rather, that of specific antibodies that can detect the epitopes of interest in a multiplexed reaction. The sensitivity, accuracy, and essentially limitless multiplexing of Abseq make it valuable for characterizing heterogeneous populations of single cells across biology.

The objective of Abseq is to enable the sensitive, accurate, and comprehensive characterization of proteins in large numbers of single cells. Cells are bound with antibodies against the different target epitopes, as in conventional immunostaining, except that the antibodies are labeled with unique sequence tags (Fig. 1a). When an antibody binds its target, the DNA tag is carried with it, allowing the presence of the target to be inferred based on the presence of the tag. In this way, counting tags provides an estimate of the different epitopes present in the cell, as detected via antibody binding.

This only allows single-cell characterization if the tag sequences belonging to one cell can be differentiated from similar tag sequences belonging to all other cells. One strategy is to perform the experiment on a single cell, as has recently been demonstrated using the NanoString technology^28^. However, to analyze large populations, a method must be employed that can pool the tags of many cells in a single sequencing reaction, but still deconvolute them according to each cell. This can be accomplished using droplet microfluidic barcoding.

Droplet barcoding has recently been described to sequence the transcriptomes of single cells or to sequence DNA molecules with deep coverage in an ultrahigh-throughput format^26^,^29^,^30^. In this approach, unique cell barcode sequences are attached to the antibody tags, creating chimeric molecules in which one portion contains the antibody identity and the other the cell identity to which it is bound. This is accomplished by isolating the antibody-bound cells (Fig. 1b) in droplets with unique cell barcode sequences (Fig. 1c), and performing PCR to link cell barcode and antibody tag sequences (Fig. 1d). In this way, all tags bound to a given cell are amplified and barcoded, providing a protein epitope profile encoded in the tag sequences of that cell (Fig. 1e).

Using droplet microfluidics, which can form, manipulate, and merge droplets at ultrahigh-throughput rates^31^,^32^, 10,000 single cells can be barcoded in under an hour. UMIs are also included in the tag sequences, allowing correction of amplification bias, and providing quantitative measures of protein abundance. To recover information on single cells, the sequence tags are bioinformatically sorted by barcode, grouping together all tags belonging to each cell. The resulting data is reminiscent of flow or mass cytometry (Fig. 1e), except that the levels of antibody binding are related not to the number of photons or mass tags impinging a detector, but rather, the number of counts of a specific tag sequence identified by the sequencer.

The number of cells that can be analyzed with Abseq is approximately *C* = *N*_*c*_ × *N*_*r*_, where *C* is the capacity of the sequencer, *N*_*c*_ the number of cells, and *N*_*r*_ the number of reads per cell. A common sequencing instrument providing a billion reads per run, would allow ~1,000 proteins to be quantified over two decades of dynamical range for over 10,000 cells. These numbers can be reduced, however, due to inefficiencies at different steps in the process, including oligo-antibody conjugation efficiency, antibody affinity and specificity, and amplification bias.

## Results

### Optimization of Abseq molecular biology

To enable single cell protein profiling, we require specific antibodies tagged with known DNA sequences. To conjugate known DNA tags to antibodies, we use bifunctional crosslinkers reactive towards thiol (via maleimide) and amine (via NHS) moieties (Fig. 2a). We conjugate 5′-thiol-modified oligonucleotides to the crosslinker via maleimide chemistry, followed by desalting. The purified oligos, now incorporating a 5′-NHS-ester, are added to a solution of antibody, where they react with amine residues on the antibody surface (Fig. 2a).

For our experiments, we use antibodies targeting surface proteins of Jurkat and Raji cancer cells. These cells are derived from the hematopoietic system, and are relatively binary in their expressions of CD3 (Jurkat) and CD19 (Raji) surface proteins. Antibodies against these targets are available commercially, which we validate for specific binding prior to DNA conjugation with fluorescence microscopy. To show the specificity of the antibodies in our model system, cells are immobilized on a glass slide using cytospin and stained with CD3 or CD19 antibodies followed by Alexa 594-labeled secondary antibodies. The fluorescence images show that the CD3 antibody binds Jurkat cells specifically, whereas the CD19 antibody binds Raji cells (Fig. 2b). To confirm successful conjugation of DNA tags to the antibodies, we perform SDS-PAGE to measure changes in molecular weight of the conjugates. The conjugated samples show shifts to higher molecular weight, indicating successful conjugation of ~54% of the antibodies (Fig. 2c).

Due to the single-molecule sensitivity of droplet PCR and DNA sequencing, it is essential to purify conjugated products, to remove unbound oligos that could cloud Abseq results. We use the Nab™ Protein A/G Spin Kit to remove unreacted oligo. The unreacted oligo runs as a low, broad band on an SDS-PAGE gel (Fig. 2d, Lane 1) while the unconjugated antibody runs as a tight and more slowly migrating band (Lane 2). The first, second, and third elutions after purification run has no or double bands (Lanes 3–5), indicating that most antibodies are conjugated. Importantly, the unreacted oligo band is absent in these elutions, indicating that they have been removed. To confirm this, we recover the contents of the purification column (flow-through) and the unreacted oligo band (Lane 6). For comparison we also provide the results for the conjugated antibody without purification, showing significant oligo contamination (Lane 7). Hence, purification is essential to provide high-quality conjugates devoid of unbound oligo for specific cell labeling.

For Abseq to yield accurate results, the DNA-conjugated antibodies must simultaneously retain their specificity for their target and efficiently deliver the oligonucleotide to the cell. To confirm binding specificity, we stain the two cell lines with the DNA-conjugated antibodies, and add FAM-labeled hybridization probes complementary to the DNA-tags. Hence, cells should only appear fluorescent if they are bound by the antibody, the antibody conjugated to the DNA tag, and the tag hybridized by the fluorescent probe. We also prepare negative controls in which we omit the fluorescent probe or antibody. We find that, as expected, when the probe or antibody is omitted, Jurkat and Raji cells exhibit minimal fluorescence (Fig. 2e), whereas when both are included, the populations shift to higher fluorescence. This demonstrates that the antibodies retain their binding affinity after conjugation.

**Figure 1:**
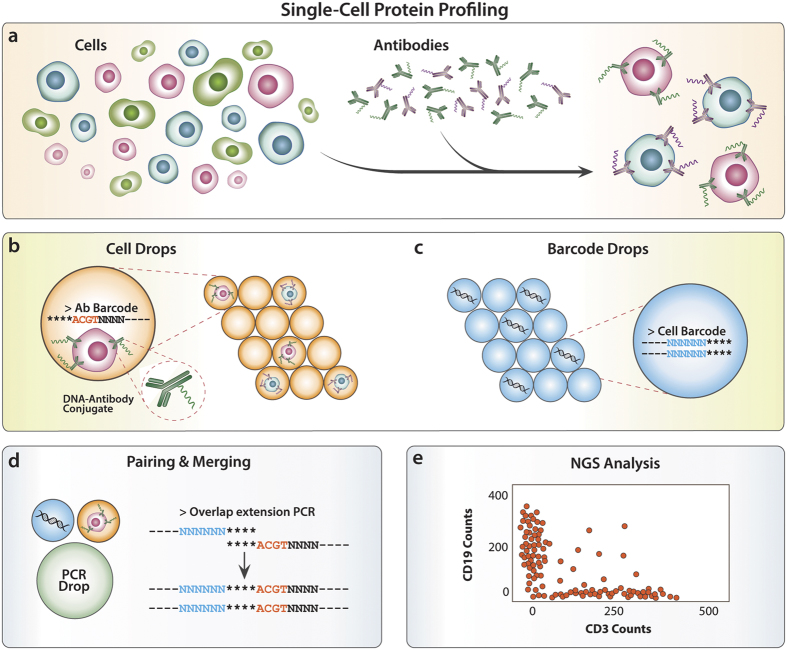
Abseq workflow (Figure edited by Sarah Pyle). Cells are stained with antibodies labeled with unique sequence tags (**a**). To read out single cell protein expression, a microfluidic workflow conjugates the antibody tag sequences bound to the cell (**b**) with unique cell barcode sequences (**c**) via splicing by overlap extension PCR (**d**). This is performed on >10,000 single cells in parallel and the chimeric products pooled and sequenced. To obtain single cell protein information, the data is sorted by barcode (**e**). Unique molecular identifiers are included to correct tag counts due to duplicated sequences resulting from PCR bias during sequencing library preparation.

**Figure 2:**
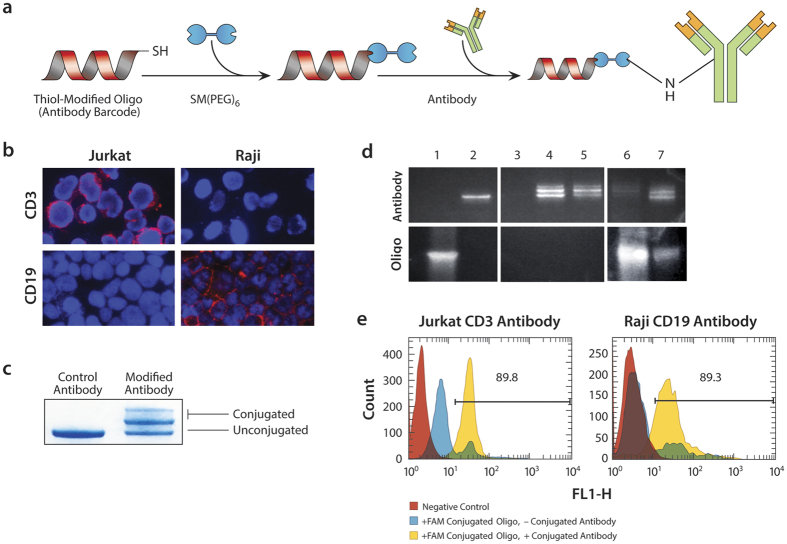
DNA-antibody conjugation. (**a**) To enable single cell protein profiling, Abseq uses antibodies labeled with known tag sequences joined via a heterobifunctional crosslinker (Figure edited by Sarah Pyle). (**b**) To confirm specificity of the antibodies, we label Jurkat and Raji cells, which are binary in their expression of CD3 and CD19, respectively, with the unconjugated antibodies, and obtain the expected labeling pattern. The images are obtained using fluorescently-labeled secondary antibodies (red) and DAPI stain for nuclear DNA (blue). (**c**) The antibody against CD3 runs as a single band when unconjugated and multiple bands after conjugation when visualized on an SDS-PAGE gel stained with SimplyBlue™ SafeStain; the shift to higher molecular weight indicates successful linkage of sequence tags to antibodies. Unbound tags remain after conjugation and must be removed, as visualized on an SDS-PAGE gel with SYBR DNA stain (**d**). Column (1) tag oligo only; (2) unconjugated CD3 antibody; (3–5) first, second and third elutions of purified fractions from Nab™ Protein A/G Spin Kit, showing that the second fraction contains most of the conjugated antibody and that oligos are successfully removed (bottom panels); (6) flow-through from column showing captured unbound oligos; (7) conjugated antibody before purification. (**e**) To confirm that the antibodies retain their affinity after conjugation, we label Jurkat and Raji cells and analyze the results with flow cytometry. We also include fluorescent oligos complimentary to the antibody tag sequences and find that fluorescence of the cells is dependent on presence of the conjugated antibodies.

**Figure 3:**
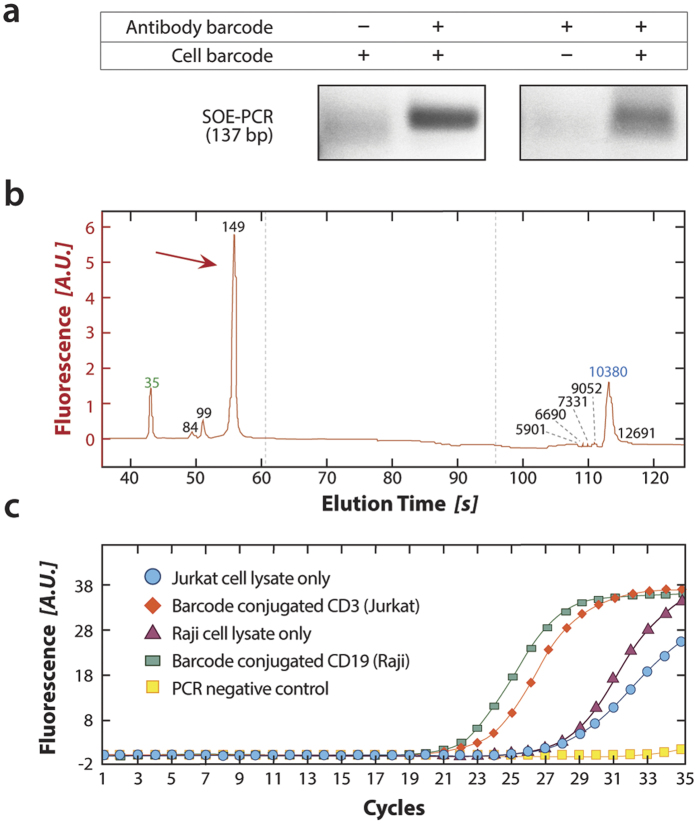
Bulk validation of SOE-PCR linkage of antibody and cell barcode sequences. (**a**) Generation of SOE-PCR product depends on the presence of both antibody tag and cell barcode sequences, as demonstrated on a 1% agarose gel stained with SYBR green. (**b**) The SOE-PCR product is pure, yielding a sharp peak on a Bioanalyzer at the anticipated molecular weight. (**c**) For Abseq to provide quantitative results, the number of SOE-PCR products must be in proportion to the number of antibody tag sequences bound to the cell, which we validate by quantitative PCR. When the appropriate antibody is used, amplification occurs early, indicating presence of much SOE-PCR product (green and red). When no or the incorrect antibody is used, amplification occurs late, indicating little SOE-PCR product.

To read out single cell antibody binding, we must splice the cell barcode sequence onto the antibody tags. The cell barcode sequence (100 bp) and antibody tag sequence (59 bp) have common homology domains (Fig. 1d) that can be utilized for strand-overlap extension PCR (SOE-PCR), producing a 139 bp linked product that can be sequenced as one read. When we perform the reaction in a tube, we obtain SOE-PCR products only when both cell barcodes and tag sequences are present (Fig. 3a). To confirm that this matches the results with cells in droplets, we repeat the experiment using the microfluidic workflow, but analyze the output with a more sensitive Bioanalyzer to quantify the results (Fig. 3b). We find that, as expected, the dominant peak is within the margin of error of the Bioanalyzer measurement (~10%), indicating successful splicing of the barcode and tag sequences. To confirm that the peak corresponds to the correct product, we recover and sequence the product and, indeed, obtain the expected sequence.

In addition to generating spliced products when cell barcode and antibody tag sequences are present, quantifying the levels of antibody binding requires that the number of these products be in proportion to the number of antibodies originally bound to the cell. To determine if this is the case, we perform a bulk reaction on millions of Jurkat (CD3^+^) or Raji (CD19^+^) cells stained with their respective conjugated antibodies, and analyze the results with quantitative PCR, which estimates the levels of SOE-PCR products in solution (Fig. 3c). As expected, when the two cell types are stained with their antibodies, much SOE-PCR product is present and the amplification begins at a low cycle number. By contrast, when the off-target or no antibody is used, little product is generated, resulting in amplification at higher cycles. This shows that expected antibody specificity is preserved after conjugation and that the quantity of SOE-PCR products reflects the number of antibodies bound to the cell. When sequencing the results, quantitation accuracy can be increased further using UMIs to correct for amplification bias of the SOE products.

### Microfluidic workflow for Abseq

To measure protein expression in single cells in an ultrahigh throughput format, we use droplet microfluidic barcoding. The microfluidic workflow consists of three devices, the first two encapsulating single cells and barcode molecules in droplets, and the third pairing one of each with a PCR droplet, to enable amplification of the tag sequences and splicing-on of the barcodes.

**Figure 4:**
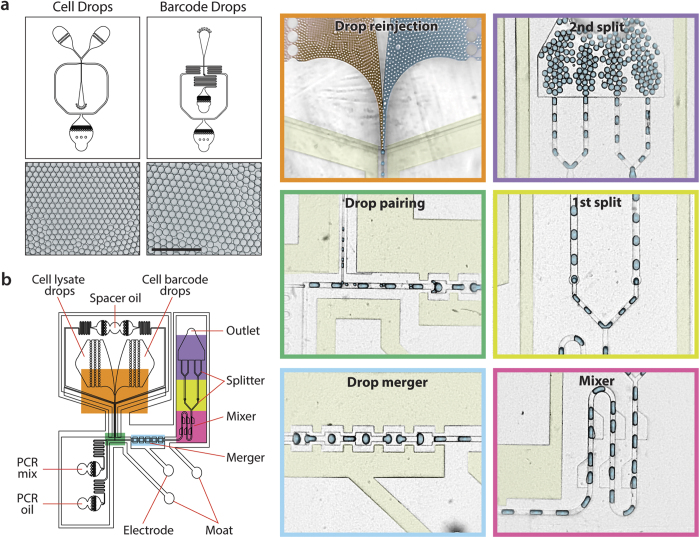
Microfluidic workflow for single cell protein profiling. (**a**) A two-inlet flow-focus droplet generator encapsulates single cells with proteinase K lysing agent into 47 μm droplets, while a one-inlet droplet maker encapsulates single barcode randomers into 53 μm droplets. (Scale Bar: 400 μm) (**b**) After thermal incubation, these droplets are controllably merged with each other and a PCR droplet using a triple-merger device. Cell and barcode droplets are introduced into two inlets, forming an interdigitated stream prior to spacing by oil (orange). One of each droplet is paired with a large PCR droplet formed upstream (green) and the three droplets electrically merged (blue). The droplets are mixed (pink) and split into two (yellow) and then four (purple) portions, followed by off-chip thermal cycling for SOE-PCR of barcodes to antibody tags.

A challenge when performing PCR on single mammalian cells in sub-nanoliter droplets, as is required in Abseq, is that cell lysate is potently inhibitory to the reaction. To overcome inhibition, we implement a workflow that digests the cells with proteinase K, heat inactivates the protease, and merges the lysate droplets with PCR reagents; this workflow, which is ultrahigh throughput, has been shown to enable efficient PCR and RT-PCR on hundreds of thousands of single cells^33^,^34^. The first step is to combine an antibody stained cell stream with a stream comprising the proteinase K lysing agent, introduced from two separate inlets on a “co-flow” microfluidic droplet maker (Fig. 4a, left). Due to the laminar flow conditions, the streams do not mix until they are encapsulated in droplets. For our flow rates 200 μL/hr for the aqueous streams, 600 μL/hr for the oil, and nozzle dimensions of 38 μm, we form 47 μm droplets at a rate of 7 million droplets per hour. Due to the speed at which the device operates, we encapsulate cells at 1 in every 30 droplets to minimize double-encapsulations, encapsulating 120,000 cells in ~30 min. The droplets are incubated at 55 °C to activate proteinase K, resulting in cell lysis, digestion of cellular proteins, and release of antibody tag sequences into the encapsulating droplet. Temperature is then increased to 95 °C for 15 min to inactivate the protease, yielding a single cell lysate emulsion ready for barcoding.

To produce the barcode emulsion, single stranded DNA of random sequence (10-mers) flanked by priming sequences, are mixed with PCR reagents and primers, and injected into a single-inlet droplet maker at a rate of ~1 molecule per 10 droplets (Fig. 4a, right). For flow rates of 300 μL/hr for the aqueous phase, 900 μL/hr for the oil phase and nozzle dimensions of 30 μm, we produce 53 μm droplets at a rate of 9 million per hour. The droplets are immediately thermocycled to amplify the single-molecule templates into a clonal population ready for barcoding.

To barcode the single-cell lysates incorporating DNA-tagged antibodies, one cell lysate and barcode droplet must be merged with one PCR droplet. The merger device consists of a PCR droplet generator followed by a triple-droplet merger junction (Fig. 4b). A side-arm combines the cell lysate and barcode emulsions into an interdigitated stream, such that the two droplet types flow in an alternating fashion (orange panel); this allows us to set flow rates such that, on average, two droplets are introduced into the main channel for each PCR droplet (green panel). The PCR droplet maker runs at flow rates of 300 μL/hr for the aqueous and 500 μL/hr for the oil which, for dimensions of 71 μm, generates 120 μm diameter droplets at a rate of ~330,000 droplets/hour. We thus set the cell lysate and barcode droplet flow rates to 30 μL/hr and 30 μL/hr, respectively, to synchronize the streams, so that three droplets are merged on average (one of each type).

To merge the three droplets, we flow them into an electrocoalescence junction consisting of a sequence of expansions and constrictions, flanked by positive and ground saltwater electrodes (blue panel). The drops are merged by passing through an alternating electric filed induced with 100–200 V. The merged droplets now contain all components for barcoding; however, the large droplets tend to be unstable to the temperatures required for PCR. To increase their stability, we mix the droplet contents using a series of “fan blade” channel expansions (pink) and split each droplet into four equal portions (yellow and purple), providing ~80 μm final droplets (250 pL) that are immediately thermocycled (11 cycles). After thermal cycling, the emulsion is broken and the DNA purified with a Zymo column, and the resultant products processed with ssDNA-specific DNase (ExoI) to remove unlinked antibody barcodes; a second round of bulk PCR is then performed to yield sufficient DNA for sequencing.

### Bioinformatic workflow for Abseq

Our bioinformatic pipeline consists of quality filtering, barcode clustering, and UMI correction of expression counts. We prepare and sequence the library using an Illumina MiSeq Kit v2 (50 cycles, single reads), providing 20 million reads. Reads are filtered by quality score provided by the sequencer and correctness of the known constant region between the tag and barcode sequences.

**Figure 5:**
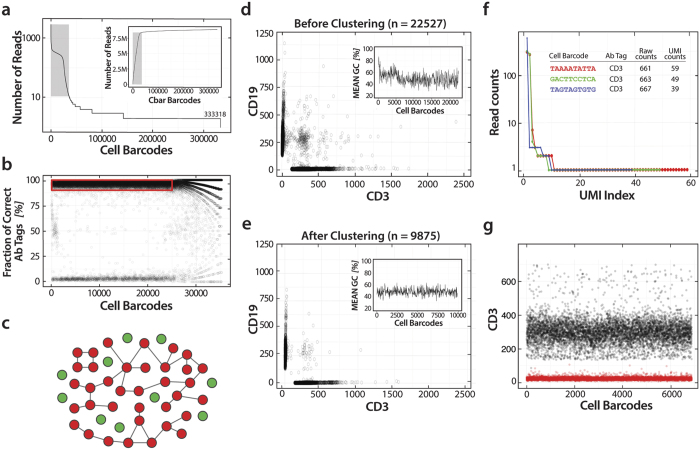
Abseq bioinformatics workflow. (**a**) To obtain high quality single cell data, the raw sequence reads are processed through quality filters. While actual barcode groups have many reads, PCR mutation generates spurious groups comprising few reads, as shown by the read count distribution for each group. 95% of reads fall within the large groups, which we select (inset). (**b**) Contaminating DNA and sequencing error also generate products that do not map to our known antibody tag sequences, as shown by plotting the fraction of reads within a group mapping to the tags versus barcode index, so we select only the groups with >90% correctly-mapping tags (red box). (**c**) PCR mutation expands a barcode sequence in Hamming space, which we thus discard (fluffy red clusters), while unmutated groups remain well isolated, which we thus keep (green points). A large number of “double positive” clusters are present (**d**) before removing groups highly connected in Hamming space that disappear when keeping only isolated groups (**e**). The insets show the GC content of the barcode groups, illustrating that many GC-rich barcode sequences are discarded by Hamming distance clustering, and implying that these groups tend to mutate and amplify faster than less GC-rich sequences. (**f**) To correct for amplification bias, UMI filtering discards duplicate tag counts evident when plotting the read count histogram for each unique UMI sequence for a specific cell for CD3. (**g**) UMI correction also reduces the noise of the measured protein profiles. Raw counts and UMI-corrected counts for CD3 tags from individual cells are shown in black and red color, respectively.

Cell barcode sequences can obtain spurious mutations due to PCR and sequencing error. Because such errors are relatively rare and our barcode sequences relatively long (10 bp), such mutations usually generate new barcode groups containing few reads. To identify and remove these spurious groups, we plot the read count distribution for all cell barcodes, sorted in descending order, along with the cumulative of this distribution (inset). The majority of reads exist within large barcode groups, as expected for true single cell data, indicating that the large number of sparsely-populated groups are artifacts due to PCR and sequencing error. We thus keep only the groups up to the inflection point (Fig. 5a, gray box) accounting for ~95% of all reads.

True single cell barcode groups should contain reads mapping only to the known antibody tag sequences used but, in practice, some groups may contain reads that map to other sequences, due to contaminating DNA or sequencing error. To remove these groups we calculate the fraction of reads within each group mapping to the known tag sequences, and plot the results as a function of barcode sequence (Fig. 5b). We find that, as expected, most barcode groups contain reads mapping to the known tag sequences (>90%) and thus select these groups for further analysis (red box). The barcode groups to the right of the plot have low read counts, resulting in an apparently quantized fraction measurement that disappears as counts increase.

If two groups happen to share similar barcode sequences, errors during barcode amplification and sequencing can create spurious groups comprising reads from both of the originally distinct groups; this, in turn, can mix reads from multiple cells in a group that appears genuine, even though it is the result of sequencing error. To remove these groups and consider only single-cell data, we analyze barcode sequences to extract “well isolated” clusters using an algorithm that compares all barcodes, clustering groups that are within one Hamming distance (Fig. 5c, red). This generates two kinds of clusters, ones containing a single barcode sequence that are not within one Hamming distance to any other barcode group, and are thus well isolated (green points), and others that contact multiple groups (red clusters). Because our barcode sequences are relatively long and we sample only ~10,000 for single-cell labeling, the likelihood of so many groups being near one another in Hamming space is small and, rather, these extended clusters are likely the result of cumulative PCR mutation. We thus identify and remove these clusters, yielding 9,875 well-isolated groups, in good agreement with our expectations based on the cell processing rates and the duration of device operation.

To confirm that this choice is justified, we exploit the fact that the cells in our study, Jurkat and Raji, are “binary” in their expression profiles of the tested markers, tending to express mostly CD3 or CD19 markers, respectively. Consequently, instances in which both markers are strongly expressed are likely artifacts of double encapsulation of cells or mergers of barcode groups due to PCR mutation. To investigate this, we plot the number of tags within each barcode group that map to the two markers before (Fig. 5d) and after (Fig. 5e) removing the highly connected clusters. Indeed, before applying this filter, there are many groups that show high expression of both markers while, after, the number is consistent with what we expect based on known rates of double cell encapsulation. Adding further evidence that these groups correspond to barcode collisions from PCR mutation is that, prior to applying this filter, there is a subset of groups with abnormally high GC content (Fig. 5d, inset) that disappears after it is applied (Fig. 5e, inset). This suggests that barcode groups with high GC content tend to mutate and amplify faster than other groups, validating the choice of discarding groups that are highly connected in Hamming space.

Sequencing instruments require a minimum input of DNA, necessitating amplification of the fused products. However, because DNA sequences can amplify at different rates, extended amplification can skew the proportions of different sequences in the library, a process known as “bias” that impacts many sequencing experiments. Single-cell sequencing is especially sensitive to bias due to the need to massively amplify the minute amount of starting material, which can skew Abseq tag counts. To correct this bias and allow more quantitative results, we implement an approach that has been developed to accurately measure gene expression profiles in single cells. We include a unique molecular identifier (UMI) sequence, consisting of 8 random nucleotides, in every antibody tag sequence. If a given tag molecule is amplified, the UMI replicates, allowing us to identify that an original template molecule has been observed more than once; this allows us to discard redundant tags, significantly enhancing the accuracy with which we can estimate the original number of tag sequences bound to the cell, and the associated protein targets.

To illustrate the value of this approach, we select three barcode groups and plot the number of times we identify the different UMI sequences in that group (Fig. 5f). While most UMIs are observed once, a small number are observed many times, due to biased PCR amplification when generating the sequencing library. If the UMIs were not present, these duplicates would inflate estimated tag counts. Indeed, by filtering counts based on UMIs, we obtain far less noisy tag count estimates (Fig. 5g).

### Ultrahigh-throughput single cell protein profiling

**Figure 6:**
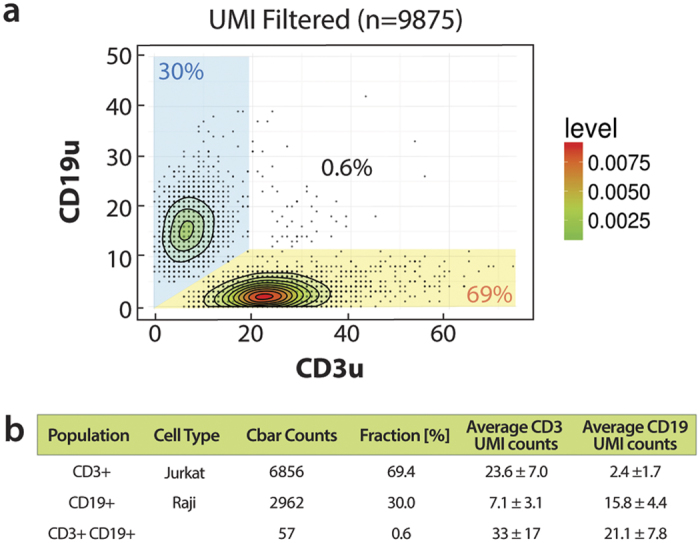
Abseq identifies T and B cell populations. (**a**) A mixed population of B and T cells are stained with a cocktail of DNA-labeled antibodies targeting CD3 and CD19 surface proteins, and the cells analyzed using Abseq. The results are presented as a scatter plot showing the number of UMI-corrected counts of tags corresponding to each marker, for every cell in the sample. Two major populations are evident that are either strongly CD3-positive (T-cells) or CD19-positive (B-cells). A third, double-positive population is also observed. (**b**) The proportions of the three populations are in approximate agreement with expectations based on the known proportions with which the cells were mixed and the probability of multiple cell encapsulations.

Our bioinformatic pipeline accepts raw, barcoded reads from the sequencer and processes them to remove low-quality sequences, spurious barcode clusters, and to correct tag counts based on UMIs. The resultant data can be used to profile proteins in single cells at ultrahigh throughput. To illustrate this, we apply the pipeline to our Jurkat and Raji cell mixed population. These two cell types are fairly binary in their expression of the surface markers CD3 and CD19 and, hence, we expect that the cells should cluster into two populations corresponding to CD3^+^CD19^−^ and CD3^−^CD19^+^. To investigate this, we plot the UMI-corrected CD3 and CD19 counts (Fig. 6a). Indeed, we observe two major populations corresponding to the anticipated Jurkat and Raji cell protein expression. We find that 69% of cells are CD3 positive and 30% CD19 positive, in agreement with the input proportions, with double-positive instances being rare (0.6%) and in rough agreement to what we expect based on the Poisson statistics for double encapsulation (Fig. 6b).

## Discussion

Abseq can profile proteins in single cells at ultrahigh throughput, and the resultant information can be used to differentiate between different cell types. Using droplet microfluidics >10,000 single cells can be profiled per experiment; while this throughput is below flow and mass cytometry, Abseq’s use of DNA tags provides a number of powerful advantages, including the ability to amplify and thereby detect low abundance tags, to correct read counts using UMIs, and essentially unlimited multiplexing potential. For example, the Helios CyTOF system (Fluidigm) can detect down to 350 antibodies/cell, and the FACSAria III Cell Sorter (BD) can detect 85 FITC molecules/particle according to manufacturers’ specification. Abseq can theoretically detect a single antibody per cell because the DNA tags are amplified from single-molecule templates. Tuning the conjugation chemistry and increasing the number of DNA tags per antibody can improve the sensitivity of Abseq as needed. Multiplexing is also important because the proteomes of most organisms are large, including multiple spliceforms and post-translational modifications of just a single gene transcript. Sequence tags allow, theoretically, unique labeling of antibodies against every member of the proteome. This, in turn, would change how experiments are performed, allowing complete and unbiased characterization of the proteome analogous to genome and transcriptome sequencing, and obviating the need to specify which biomarkers to target *a priori*. Instead, relevant biomarkers would be learned from the data, maximizing the chances of unexpected discoveries.

The principal limitation to throughput and sensitivity of Abseq is sequencing depth. For example, assuming 10,000 cells are analyzed, and 10 antibodies are probed, a standard MiSeq run of ~20 million reads provides a dynamic range of 200 counts per antibody per cell. This, in practice is lowered by inefficiencies in the process such as loss of DNA during barcoding and bias introduced during sequencing preparation. In our analysis of Jurkat and Raji cells, estimated with quantitative flow cytometry to have 20,000 and 37,000 copies of CD3 and CD19, respectively, our small MiSeq run of ~3 million quality-filtered reads for ~10,000 cells yields ~300 reads per cell. UMI-filtering to remove PCR duplicates yields ~20 unique UMI counts per cell, on average. While this detects only a small fraction of the proteins present, it allows ratios between different protein expressions to be measured and, thus, clustering and identification of different cell types. Moreover, in most cases of interest, it is unnecessary to detect every protein present in a cell and, rather, the goal is to provide the expression profiles of many proteins for each cell. In this case, the depth per antibody can be reduced, allowing the available reads to be spread across more antibodies and cells. If higher sensitivity is desired, the number of cells analyzed can be decreased and the sequencing capacity increased. For example, for 1,000 cells and using a HiSeq run providing ~1 billion reads, 10 proteins can be quantitated over a range of 100,000 counts, or ~5 decades, which is comparable to current flow cytometers. Thus, increases in sequencing capacity will continue to enhance the sensitivity and throughput of Abseq, while the ability to assign unique DNA tags to each antibody already provides a fundamental advantage in multiplexing power.

If sequencing depth were not limiting, the cost of the droplet PCR reactions and sequencing library preparation would be. While the device indeed requires a 2:1 droplet fusion, this is nevertheless extremely fast for a well-designed device, allowing us to merge droplets at >500/second. For cell and barcoding loading rates of ~10%, this provides ~5 usefully barcoded cells per second, which barcodes 10,000 cells in under an hour. To make the droplet PCR efficient, the droplet volume and PCR reagent concentrations are critical, and the requisite 1 million droplets consume ~1 mL of PCR reagent. While this is an acceptable consumption, it becomes prohibitively expensive to go much higher without improvements to the microfluidics to enable use of smaller droplets. However, there are examples for single cell PCR reactions in droplets as small as ~60 pL^35^, which would allow 10,000 cells to be barcoded using ~60 μL of reagent and, thus, 200,000 cells using ~1.2 mL of reagent. In addition, swapping the droplet barcodes with hydrogel barcodes, the barcode Poisson encapsulation can be removed^36^, so that every cell is paired with a barcoded hydrogel, which is now incorporated into commercial droplet barcoding systems^37^. This removes one of the Poisson factors so that essentially every cell would be paired with a barcode, allowing ~100,000 cells to be barcoded using our current droplet volumes and ~2 million cells using 60 pL droplets. Thus, if sequencing is not limiting, there is much potential to increase the throughput of Abseq.

An additional limitation of Abseq, like all other methods relying on antibodies, is that the antibodies must have high specificity and affinity, and must be available. While antibody generation is non-trivial, new techniques and commercial sources should improve quality and availability in future years. Nevertheless, even with this challenge, the current limit to multiplexing is often the inability of labeling existing antibodies with uniquely detectable tags^17^,^38^. Abseq addresses this, allowing effectively limitless multiplexing and shifts the burden back to antibody generation and conjugation.

While we have applied Abseq to surface proteins, it should be extendable to internally-expressed markers too, since antibodies can bind intracellular targets after permeabilization. The microfluidic method for lysing and barcoding the cells utilizes proteases that digest and solubilize even fixed cells and tissues, freeing the antibody tags for barcoding. This should enable new opportunities for applying Abseq to directly detect, for example, the phosphorylation state of proteins in pathways of interest without having to know how they correlate with cell surface expression. Abseq should also enable profiling of cell state, by detecting post-translational modification of proteins in relevant cell pathways.

The droplet barcoding method utilized by Abseq is similar to ones recently applied to barcode DNA molecules and the transcriptomes of single cells^29^,^30^, holding the exciting possibility of combining all into a single workflow. This would allow simultaneous characterization of genome, transcriptome, and proteome for large populations of single cells, to track the flow of information through the Central Dogma of biology. This would be valuable for studying heterogeneous systems across biology, including the development of myriad tissues and the progression of cancer.

## Methods

### Cell culture

Jurkat cells (clone E6-1) and Raji cells were cultured in RPMI-1640 medium supplemented with 5% fetal bovine serum (FBS) and antibiotics. The cells were cultured at 37 °C in the presence of 5% CO_2_.

### Antibody conjugation and purification

Thiol modified oligonucleotides were reduced by adding 10 μL of 1 M DTT per 10 nmol of lyophilized DNA to a final concentration of 100 μM. 15 μL (100 μM) of reduced oligos were purified using an oligo clean concentrator kit (Zymo Research) and eluted in 15 μL of water. Purified oligos were treated with 1.5 μL of 10X PBS pH 7.2 and 0.5 μL of 2.5 mM SM(PEG)_6_ for 20 min at room temperature. Oligos were purified once again using an oligo clean concentrator kit and eluted in 15 μL of water. 1.5 μL of 100 mM HEPES pH 8.2 was added to the oligos. 10 μL of antibody (1 μg/μL) was added to the oligos and allowed to incubate at room temperature for 30 min. Antibody purification was performed using the Nab™ Protein A/G Spin Kit (Thermo Scientific) following the protocol.

### Immunostaining

5 × 10^4^ cells were washed with 1 ml of cold PBS and resuspended in 100 μL of cold PBS containing 40% FBS. Cells were transferred to a glass slide via cytospin. Cells were briefly dried and fixed with 4% paraformaldehyde for 20 min at room temperature. Cells were washed with cold PBS and blocked with PBS containing 10% goat serum for >1 hour at room temperature. Cells were stained with 200 μL of primary antibody diluted in PBS containing 5% goat serum and incubated overnight at 4 °C. Cells were washed with PBS and incubated with 200 μL of the secondary antibody for 1 hour at room temperature in the dark. Cells were washed and treated with Vectashield mounting medium containing DAPI (Vector Laboratories). The antibodies were LEAF Purified anti-human CD3 antibody (BioLegend), LEAF Purified anti-human CD19 antibody (BioLegend), Alexa Fluor 546 goat anti-mouse (Molecular Probes).

### FACS analysis

For each sample to be stained with conjugated antibody and analyzed via FACS, 1 × 10^7^ cells were washed with 1 mL of cold PBS and blocked with 500 μL of blocking buffer on ice for 30 min. The blocking buffer consisted of 2% FBS, 2% goat serum, 2% mouse serum, and 1 μg/mL double stranded salmon sperm. Cells were stained with 1 μg of antibody for 30 min on ice. Cells were washed three times with 3 mL of PBS plus 2% FBS (FACS buffer). 300 μM of FAM-conjugated single stranded oligos targeted to the antibody-barcode homology domain sequence was added to the stained cells in 100 μL of FACS buffer. Cells were incubated 20 min at room temperature in the dark. Cells were washed three times with cold FACS buffer and analyzed with a BD FACSCalibur (BD Biosciences). To estimate protein copy numbers, Jurkat and Raji cells were stained with FITC-labeled CD3 and CD19 antibodies (BioLegend FITC Anti-Human CD3 #300406 and FITC Anti-Human CD19 #302206), respectively, using the same staining protocol as the cells for droplet analysis. The average fluorescence intensities were fitted to the calibration curve generated from fluorescently labeled microspheres measurements (Bangs Laboratories, Quantum FITC-5 MESF) following the manufacturer’s protocol.

### Cell staining and in droplet cell lysis

5.0 × 10^5^ of Jurkat and Raji cells were collected mixed and washed with cold PBS plus 2% FBS (FACS buffer). Cells were incubated in 1 ml of blocking buffer containing 2% FBS, 2% goat serum, 2% mouse serum, and 1 μg/mL double stranded salmon sperm DNA on ice for 30 min. Cells were centrifuged at 300 RCF for 3 min and resuspended in 100 μL of blocking buffer containing 2.5 μg of Human BD Fc Block (BD Biosciences) for 10 min. at room temperature. 1 μg of each conjugated antibody was added to the cells and the cells were incubated on ice for 30 min. Cells were washed five times with 1 mL of FACS buffer. Cells were resuspended in 100 μL of FACS buffer containing 17% Optiprep. Lysis buffer containing 100 mM Tris pH 8.0, 2% Tween 20 and 1.5 μg/μL Proteinase K (New England BioLabs) was introduced to the cells via a co-flow device. Drops were generated in HFE 7500 fluorinated oil (3M) containing 2% PEG-PFPE amphiphilic block copolymer surfactant (Ran Biotechnologies). Cells were lysed via Proteinase K at 55 °C for 30 min. Proteinase K was inactivated by incubation at 95 °C for 15 min.

### Device fabrication

The microfluidic devices were fabricated using soft lithography^39^. SU-8 3025 photoresist (MicroChem) was used to make a 24-μm-tall master mold structure on a 3-inch silicon wafer using standard photolithography techniques. PDMS prepolymer (Momentive, RTV 615) mixed with curing agent at 10:1 ratio was poured onto the master placed in a petri dish. After degassing under vacuum, the PDMS was cured at 65 °C for 1 hour and cut out. Holes were punched at inlet and outlet ports using a 0.75 mm biopsy punch (Harris, Uni-Core 0.75). After cleaning with scotch tape, the PDMS channel structure was bonded to a glass substrate by treating with oxygen plasma for 60 s at 1 mbar in a plasma cleaner (Harrick Plasma, PDC-001). The channel surface was treated with Aquapel to render it hydrophobic.

### Droplet SOE-PCR and Droplet formation

SOE-PCR analysis was performed using Illumina primers: P5: 5′ AAT GAT ACG GCG ACC ACC GA 3′ and P7: 5′ CAA GCA GAA GAC GGC ATA CGA GAT 3′. FAM-conjugated probe 3Taq1: 5′ AGA TCG GAA GAG CGT CGT GTA GG 3′ was targeted to the homology domain shared by the antibody and cell barcodes. qPCR was performed using Platinum Multiplex PCR Master Mix (Thermo Fisher Scientific), 2% w/v Tween 20, 2% w/v PEG 6000 and Mx3005P qPCR System (Agilent Technologies). Drops were generated in HFE 7500 fluorinated oil (3M) containing 2% PEG-PFPE amphiphilic block copolymer surfactant (Ran Biotechnologies). Prior to PCR reaction oil exchanged was performed using fluorinated oil (FC40) with 5% PEG-PFPE amphiphilic block copolymer. PCR parameters as follows: 95 °C for 2 min, 11 cycles of 95 °C for 30 seconds, 60 °C for 90 seconds, 72 °C for 20 seconds followed by final extension at 72 °C for 10 min. Droplets were coalesced by adding 100 μL perfluorooctanol (Sigma, 370533) and centrifuging at 1000 g for 1 minute. The aqueous phase was transferred to a spin column for purification (Zymo Research, DNA Clean & Concentrator).

### Library preparation and NGS

The purified DNA was quantified with fluorescence (Thermo Fisher Scientific, Qubit dsDNA HS Assay Kit). The library was characterized with a Bioanalyzer (Agilent, High Sensitivity DNA Analysis Kit) and quantified with qPCR (New England Biolabs, NEBNext Library Quant Kit for Illumina). A 15 pM library concentration was used for the NGS runs on the MiSeq sequencer (Illumina).

### Bioinformatics

The obtained Fastq files were analyzed using the R language version 3.3.1 (ShortRead 1.30.0 package). The raw reads were first filtered by eliminating ones having more than two mutations in the homology region (sequence: GCAGTGGTATCAACGCAGAG) using agrep function. The filtered reads were sorted by Cell Barcodes (sequence: NNNN NNNN) and their read counts in descending order using the tables function. The antibody tag counts were calculated by matching to CD3 (TTATAAC) and CD19 (TTAATTG) tag sequences and corrected by eliminating duplicate copies according to UMI counts. For antibody tag and UMI counting, the fmatch function of fastmatch 1.0 package was used. The Hamming distance filtering (Fig. 5c) was performed using custom Python code based on a depth-first search algorithm. All source code will be available upon request.

## Additional Information

**How to cite this article**: Shahi, P. *et al*. Abseq: Ultrahigh-throughput single cell protein profiling with droplet microfluidic barcoding. *Sci. Rep.* **7**, 44447; doi: 10.1038/srep44447 (2017).

**Publisher's note:** Springer Nature remains neutral with regard to jurisdictional claims in published maps and institutional affiliations.
